# Role of Parkin-mediated mitophagy in the protective effect of polydatin in sepsis-induced acute kidney injury

**DOI:** 10.1186/s12967-020-02283-2

**Published:** 2020-03-04

**Authors:** Youguang Gao, Xingui Dai, Yunfeng Li, Guicheng Li, Xianzhong Lin, Chenmu Ai, Yuanyuan Cao, Tao Li, Bo Lin

**Affiliations:** 1 Department of Anaesthesiology, The First Affiliated Hospital of Fujian Medical University, 20 Chazhong Road, Fuzhou, 350005 Fujian China; 2 Department of Critical Care Medicine, The First People’s Hospital of Chenzhou/Affiliated Chenzhou Hospital, Southern Medical University, No. 102 Luojiajing, Chenzhou, 423000 China

**Keywords:** Polydatin, Acute kidney injury, Mitophagy, Mitochondria, Inflammasome

## Abstract

**Background:**

We have reported that polydatin (PD) alleviates mitochondrial dysfunction in rat models of sepsis-induced acute kidney injury (SI-AKI), but the mechanism is not well understood. Here, we investigated the role of Parkin-mediated mitophagy in the protective effects of PD in SI-AKI in mice.

**Methods:**

Sepsis was induced in the mice by caecal ligation and puncture. Mitophagy was determined by mitochondrial mass. NLRP3 inflammasome activation was determined by NLRP3, ASC and caspase-1. Mitophagy was blocked by treatment with mitochondrial division inhibitor-1 and Parkin knockout.

**Key results:**

PD treatment increased the sepsis-induced loss of mitochondrial mass, indicating the upregulation of mitophagy. Furthermore, PD treatment mediated Parkin translocation from the cytoplasm to the mitochondria. This suggests that Parkin-mediated mitophagy is an underlying mechanism. This was confirmed by the suppression of PD-induced mitophagy in Parkin−/− mice and in mice that were treated with a mitophagy inhibitor. PD-induced Parkin translocation and mitophagy were blocked by inhibiting SIRT1; thus, activation of SIRT1 might be an important molecular mechanism that is triggered by PD. Additionally, PD treatment protected against sepsis-induced kidney injury. These effects were blocked by inhibition of Parkin-dependent mitophagy. Furthermore, PD also protected against mitochondrial dysfunction and mitochondria-dependent apoptosis, and the effect was blocked when Parkin-dependent mitophagy was inhibited. Finally, PD suppressed NLRP3 inflammasome activation that was also dependent on Parkin-mediated mitophagy.

**Conclusions:**

These findings indicate that Parkin-mediated mitophagy is important for the protective effect of PD in SI-AKI, and the underlying mechanisms include the inhibition of mitochondrial dysfunction and NLRP3 inflammasome activation.

## Background

Acute kidney injury (AKI) is a known feature of severe sepsis and is associated with high mortality rates [1–3]. Although antibiotics may treat the source of sepsis, no specific therapy is available for the associated organ injury. Mitochondrial dysfunction (MD), which occurs in many pathophysiological conditions, acts as a major contributor to the development of sepsis-induced AKI (SI-AKI) [1, 4]. The key to the optimal treatment of SI-AKI lies in targeting MD. However, currently, there are no methods that effectively alleviate or halt the progression of MD in AKI.

Mitophagy is a key cytoprotective mechanism that involves the removal of impaired mitochondria and preservation of a healthy population of mitochondria. Mitophagy plays an important role in maintaining a healthy population of mitochondria by ensuring that there is an optimal balance between mitochondrial biogenesis and turnover [5, 6]. Recently, the involvement of Parkin-mediated mitophagy in nephropathy has come to light [7, 8]. In this mitophagy process, Parkin, which is an E3 ubiquitin ligase, is transported from the cytoplasm to depolarized mitochondria, where it subsequently induces the autophagic degradation of dysfunctional mitochondria. A recent study demonstrated that Parkin-mediated mitophagy protects renal tubular epithelial cells from cisplatin-induced injury [9]. Additionally, the role of mitophagy in AKI is also gaining importance [10, 11]. However, the role of Parkin-mediated mitophagy in SI-AKI has not been reported thus far.

The NACHT, LRR, and PYD domain-containing protein 3 (NLRP3) inflammasome is an important component of the innate immune system, as it initiates early inflammatory responses by inducing caspase-1 activation and IL-1β maturation [10, 11]. The NLRP3 inflammasome plays a role in the pathogenesis of renal diseases, including AKI [12]. One study showed that knockout of the NLRP3 inflammasome protected mice against ischaemia-induced AKI [13]. Furthermore, inhibition of the NLRP3 signalling pathway protects against sepsis-induced AKI [14]. In recent years, the role of mitophagy in the regulation of NLRP3 activation has been the focus of several studies [15–17]. These studies reported that mitophagy is a negative regulator of NLRP3 inflammasome activation [17], and Parkin-mediated mitophagy decreases NLRP3 inflammasome activation and protects against contrast-induced AKI [16]. Based on these findings, it would also be interesting to explore whether this mechanism of Parkin-mediated mitophagy occurs in SI-AKI.

Our previous study demonstrated that polydatin (PD) attenuates MD and inflammation in a rat model of SI-AKI [1], but the underlying pathways were not investigated. We have also demonstrated in a previous study that PD mediates Parkin-dependent mitophagy and protects against acute respiratory distress syndrome [18]. Based on our previous findings and those of other studies, in the present study, we hypothesized that Parkin-mediated mitophagy plays a role in the protective effects of PD against MD and NLRP3 activation in SI-AKI and established a mouse model of sepsis. This is also the first study to provide evidence for the role of Parkin-mediated mitophagy in SI-AKI.

## Materials and methods

### Materials

For extraction of mitochondrial and cytosolic proteins, a commercially available kit from Sigma-Aldrich (#MITOISO2, Saint Louis, MO, USA) was used. For staining of mitochondria, MitoProbe™ JC-1 (5,5ʹ,6,6ʹ-tetrachloro-1,1ʹ,3,3ʹ-tetraethyl-imidacarbocyanine iodide) (#M34152, Molecular Probes, Invitrogen, CA) was used. A luciferase-based assay kit was purchased from Promega Corp. (#G7570, Madison, WI). Parkin (#ab77924), TOM20 (#ab186735), TIM23 (#ab230253), PGC-1α (#ab191838), mt-TFA (#ab131607), LC3 (#ab51520), P62 (#ab56416), P62 phospho S349 (#ab211324), COX IV (#ab202554), NLRP3 (#ab214185), ASC (#ab175449), caspase-1 (#ab179515), cleaved caspase-3 (#ab49822), IL-1β (#ab9722), KIM-1 (#ab47635), Bax (#ab32503), Bcl-2 (#ab182858) and GAPDH (#ab181602) antibodies were purchased from Abcam (Cambridge, UK). The CellTiter-Glo assay and terminal deoxynucleotidyl transferase dUTP nick-end labelling (TUNEL) staining kit were obtained from Keygen Biotech (#KGA7037, Nanjing, China). ELISA kits to assay inflammatory cytokines (tumour necrosis factor-α [TNF-α, #1217202], interleukin-1β [IL-1β, #1210122], and interleukin-6 [IL-6, #1210602]) were obtained from Dakewe Biotech (Shenzhen, Guangdong, China). The immunohistochemical kits were obtained from Beyotime (#P0203, Shanghai, China), and all the other chemicals were purchased from Sigma-Aldrich (Saint Louis, MO, USA).

### Establishment of a sepsis model by caecal ligation and puncture

Adult male and female C57BL/6 mice (body weight, 22–25 g) were obtained from the Experimental Animal Centre of Fujian Medical University, Fuzhou, China. Parkin−/− mice (on a C57BL/6 background) were from Cyagen Biosciences Inc. (Nanjing, China). The animals were given ad libitum access to food and water and were used for the experiments only after a 1-week acclimatization period. The experiments conducted in this study received the approval of the Animal Care and Use Committee of Fujian Medical University, Fuzhou, China. The animals were treated in a manner that was compliant with the guidelines of the National Institutes of Health and the Chinese National Guidelines.

Sepsis was induced by caecal ligation and puncture (CLP). As previously described [19], a midline incision was made with minimal dissection, and caecal ligation was performed just below the ileocaecal valve to maintain intestinal continuity. Then, a 7-gauge needle was used to make a single perforation in the caecum, which was gently pressed until all the faeces were drained. The bowel was placed back in the abdomen, and the incision was closed. The same procedure was performed in the control group, but without ligation or puncture of the caecum. After the procedure, the mice were not fed, but they were given free access to water. Immediately after the CLP procedure, the mice were administered 30 mg/kg PD (or mitochondrial division inhibitor [mdivi-1], 3 mg/kg) and EX527 (10 mg/g) via tail vein injection, based on our previous findings [1, 18, 20], and they were sacrificed at 12 h after CLP. In the present study, 6 mice were included in each group.

### Histopathological and immunohistochemical analyses

Kidney sections were fixed in 10% buffered formalin, subjected to paraffin embedding, and stained with haematoxylin and eosin (H&E). The sections were evaluated for the loss of the brush border, tubular dilation, cast formation, and cell lysis. The observers were blinded to the experimental groups, and the sections were scored based on the damage visible in the tubules: a score of 0 was assigned if there was no damage; 1 was assigned if there was < 25% damage; 2, if there was 25–50% damage; 3, if there was 50–75% damage; and 4, if there was 75% damage. A minimum of 10 high-power fields (400×) were scored for each of the mice.

The sections were immunohistochemically stained with anti-NLRP3 (1:200 dilution), anti-ACS (1:200 dilution) and anti-caspase-1 antibodies (1:200 dilution) overnight at 4 °C. An avidin–biotin–peroxidase complex kit was used for immunostaining, and haematoxylin was used for counterstaining.

### Western blot analysis

Homogenization of renal tissue was carried out in ice-cold tissue lysis buffer. This was followed by pyrolysis for 30 min and centrifugation at 10,000×*g* for 20 min at 4 °C. Protein was extracted from the cytosolic and mitochondrial fractions with an isolation kit according to the manufacturer’s instructions. The protein concentration was measured by the bicinchoninic acid (BCA) method. The protein fractions were separated using SDS-PAGE (8% gel for NLRP6 and PGC-1α, 14% gel for the other proteins), and the blots were transferred to PVDF membranes for immunoblotting. The membranes were blocked with 5% skimmed milk at room temperature for 2 h. This was followed by overnight incubation at 4 °C with monoclonal antibodies against TOM20 (1:1000 dilution), TIM23 (1:1000 dilution), PGC-1α (1:1000 dilution), mt-TFA (1:1000 dilution), Parkin (1:1000 dilution), LC3 (1:1000 dilution), P62 (1:1000 dilution), phospho P62 S349 (1:500 dilution), NLRP3 (1:1000 dilution), ASC (1:1000 dilution), Caspase-1 (1:1000 dilution), IL-1β (1:1000 dilution), KIM-1 (1:1000 dilution), cleaved caspase-3 (1:1000 dilution), Bax (1:1000 dilution) and Bcl-2 (1:1000 dilution). The membranes were then incubated with the corresponding secondary antibodies (1:5000 dilution) at room temperature for 2 h. Immunoreactivity was measured using an enhanced chemiluminescence detection system (Beyotime, Haimen, China) and radiographically visualized (Kodak, Shanghai, China). GAPDH (1: 5000 dilution) and COX IV (1: 2000 dilution) were used as controls for analysis of the cytoplasmic and mitochondrial fractions, respectively.

### Isolation of renal tubular epithelial cells

Renal tubular epithelial cells (RTECs) were isolated from renal tissue using our previously published methods [1]. The renal cortex was cut into fragments and incubated with 1 mg/mL type-I collagenase for 30 min at 37 °C to induce cell dissociation. Red blood cells were removed by lysis. Finally, RTECs were collected using Percoll density-gradient centrifugation.

### Measurement of mitochondrial membrane potential

Mitochondrial membrane potential (MMP) was measured by incubating RTECs with the potential-sensitive fluorescent dye JC-1 (5 μmol/L) for 15 min at 37 °C. The treated cells were observed under a confocal microscope (LSM780; Zeiss Microsystems, Jena, Germany).

### Measurement of cellular ATP

Intracellular ATP was measured using a luciferase-based assay, as described by the manufacturer. The isolated RTECs (in a 100-μL suspension, at a concentration of 10,000 cells per well) were incubated with 100 μL of CellTiter-Glo^®^ at room temperature for 10 min to ensure stabilization of the luminescence signal. Luminescence was measured with an automatic microplate reader (SpectraMax M5; Molecular Devices, Sunnyvale, CA).

### TUNEL staining

Apoptosis of the isolated RTECs was assessed using TUNEL staining. The cell nuclei were stained with Hoechst. Apoptotic cells were visualized based on the emission of green fluorescence under 100× magnification. The apoptotic index was defined as the mean proportion of TUNEL-positive cells in 10 random visual fields.

### Serum creatinine and cytokine measurement

Serum creatinine was assayed using an AU680 automatic biochemical analyser (Beckman Coulter, Brea, CA). Serum TNF-α, interleukin (IL)-1β and IL-6 (IL-6) and IL-1β in the kidney were measured using commercially available ELISA kits according to the manufacturer’s recommendations.

### Statistical analysis

The data are shown as the mean ± S.D. Between-group differences were determined using one-way ANOVA, and the LSD multiple-comparison test and Student’s *t*-test were used as appropriate. A value of P < 0.05 was considered to indicate statistical significance.

## Results

### PD-induced mitophagy in SI-AKI

**Fig. 1:**
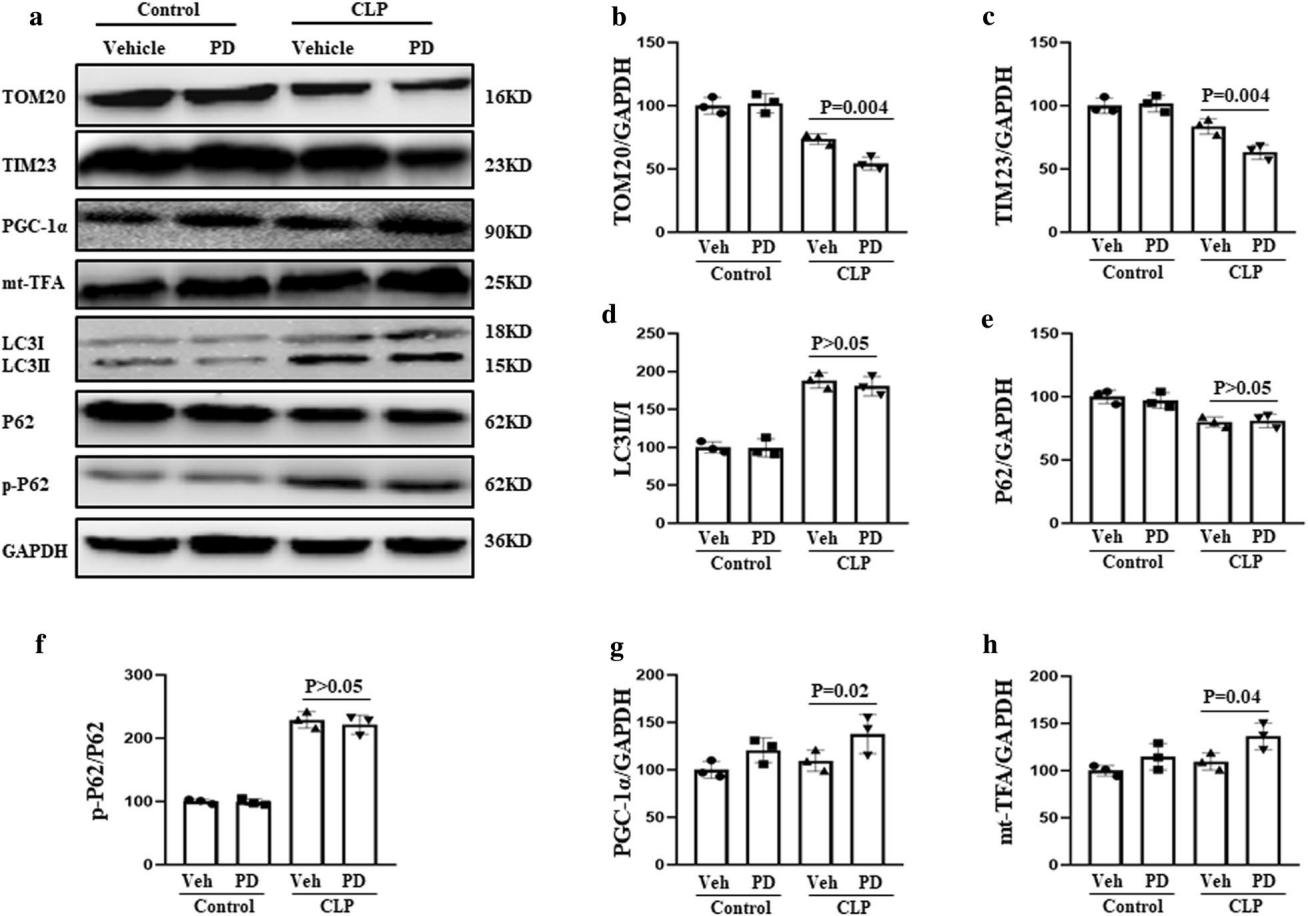
PD-induced mitophagy in SI-AKI model mice. The mice were subjected to CLP, administered PD (30 mg/kg) or vehicle, and sacrificed at 12 h after CLP. **a** Western blot analysis of the following proteins was performed: TOM20 and TIM23 (mitochondrial markers), PGC-1α and mtTFA (mitochondrial biogenesis markers), and LC3II/I, P62 and phosphorylated P62 (autophagy-associated proteins). Quantification of **b** TOM20, **c** TIM23, **d** LC3II/I, **e** P62, **f** P62 phosphorylation, **g** PGC-1α, and **h** mt-TFA

Mice that were subjected to CLP (to induce sepsis) were treated with PD (30 mg/kg) or vehicle (DMSO) and sacrificed at 12 h after CLP. The mitochondrial proteins TOM20 and TIM23 were used as mitophagy markers. PD treatment resulted in a significant decrease in TOM20 and TIM23 levels in SI-AKI mice (Fig. 1). This effect may be the result of mitophagy; alternatively, it could be the result of an increase in autophagy or decrease in mitochondrial biogenesis. Therefore, the expression of two autophagy-related proteins, LC3 and p62, as well as two mitochondrial biogenesis-related proteins, PGC-1α and mt-TFA, was analysed. PGC-1α and mt-TFA expression levels were not significantly downregulated after PD treatment. Moreover, PD did not result in significant upregulation of LC3-II or phosphorylation of P62 or significant downregulation of P62. These findings indicate that the effects of PD do not involve autophagic mechanisms. Thus, it can be deduced that PD upregulates mitophagy in mice with SI-AKI.

### Role of Parkin in PD-induced mitophagy

**Fig. 2:**
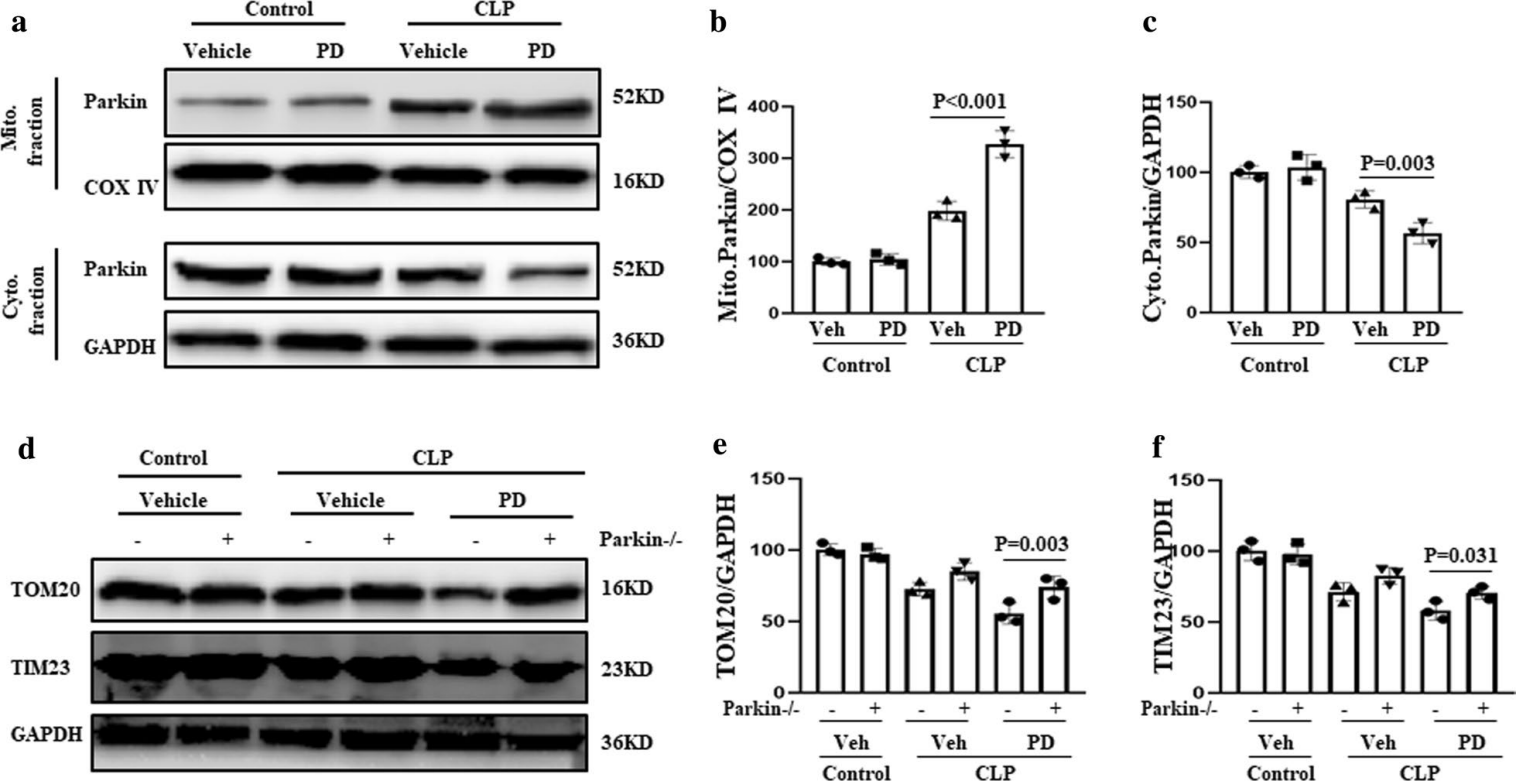
Role of Parkin in the activation of PD-induced mitophagy. **a** The mice were subjected to CLP, administered PD (30 mg/kg) or vehicle, and sacrificed at 12 h after CLP. Parkin levels in the mitochondrial and cytosolic fractions of renal cells were evaluated by western blot analysis. Quantification of **b** mitochondrial and **c** cytoplasmic Parkin levels. **d** Wild-type and Parkin−/− mice were subjected to CLP and administered PD or vehicle for 12 h. Western blot analysis of the mitochondrial markers TOM20 and TIM23 was performed. Quantification of **e** TOM20 and **f** TIM23

Parkin-mediated mitophagy is known to involve the translocation of Parkin from the cytosol to the mitochondria. Therefore, in this study, mitochondrial and cytosolic fractions of renal cells were separated and analysed for the presence of Parkin. PD treatment resulted in an increase in the level of Parkin in mitochondria, which was accompanied by a decrease in the cytosolic levels of Parkin in the treated mice compared with those of the control mice (Fig. 2a–c). These findings indicate that PD promoted the translocation of Parkin from the cytoplasm to the mitochondria in SI-AKI.

To confirm the involvement of Parkin in PD-mediated mitophagy, mitophagy activity was examined in Parkin−/− mice. Wild-type or Parkin−/− mice were established as a model of SI-AKI by CLP, and they were treated with PD (30 mg/kg) or vehicle for 12 h. The levels of the mitochondrial markers TOM20 and TIM23 were significantly higher in the PD-treated Parkin−/− mice than in the PD-treated wild-type mice (Fig. 2d–f). These findings suggest that PD-mediated mitophagy was inhibited in the absence of Parkin. Thus, PD treatment increases Parkin-dependent mitophagy in SI-AKI.

### Role of Sirtuin-1 in PD-induced mitophagy

**Fig. 3:**
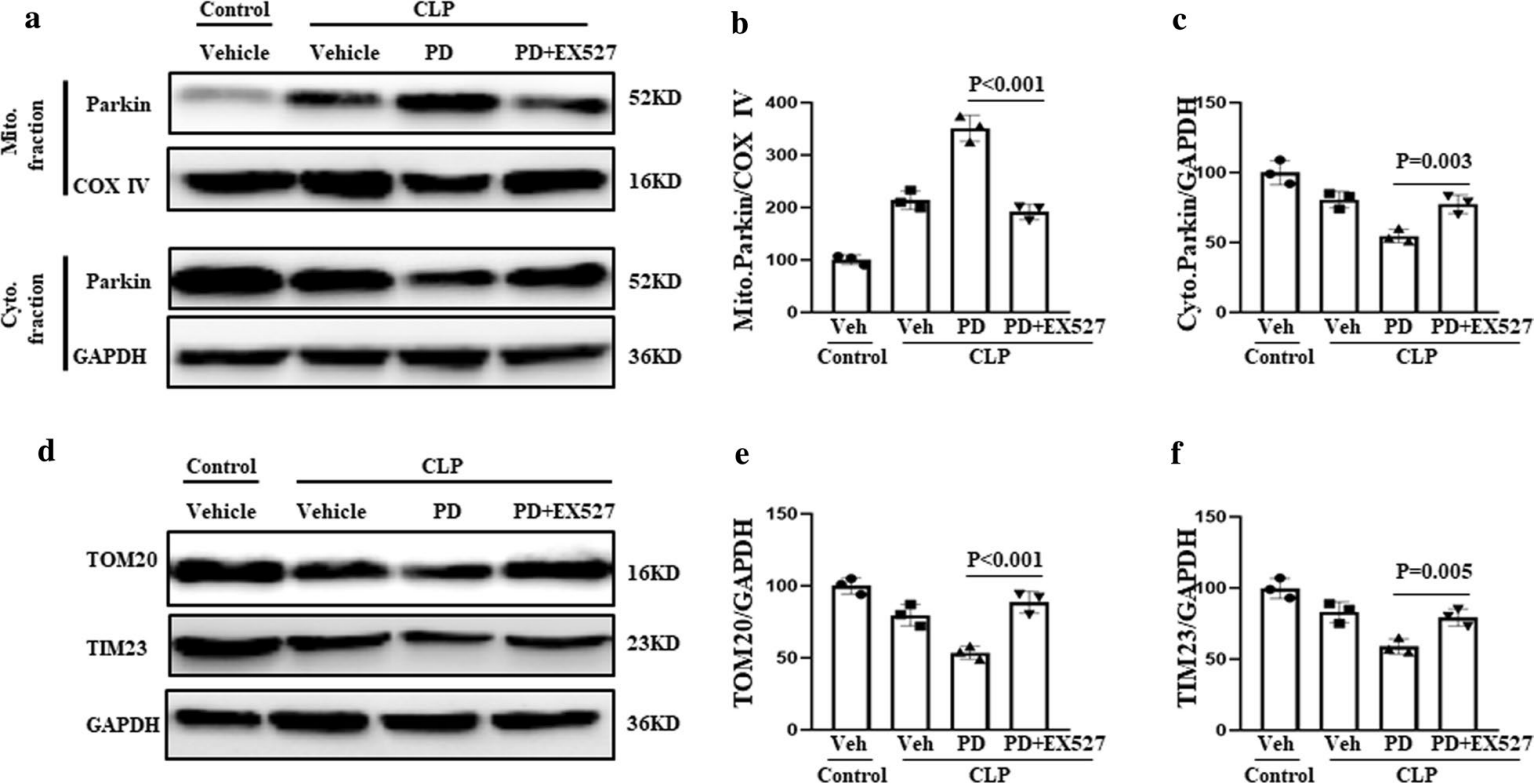
Induction of mitophagy by PD via activation of SIRT1. The mice were subjected to CLP, administered vehicle, PD (30 mg/kg), or PD plus EX527 (10 mg/kg). **a** Parkin levels in the mitochondrial and cytosolic fractions of renal cells were assessed by western blot analysis. Quantification of **b** mitochondrial and **c** cytoplasmic Parkin levels. **d** The levels of the mitochondrial markers TOM20 and TIM23 were measured by western blot analysis. Quantification of **e** TOM20 and **f** TIM23

Sirtuin (SIRT1) plays an important role in the regulation of Parkin-mediated mitophagy [21]. To determine whether SIRT1 also plays a role in Parkin-mediated mitophagy in the context of SI-AKI, we treated renal cells from SI-AKI model mice with EX527, an inhibitor of SIRT1. The mice were administered vehicle, PD (30 mg/kg), or PD plus EX527 (10 mg/kg). We found that PD-induced downregulation of TOM20 and TIM23 was decreased in mice that received both PD and EX527 treatment (Fig. 3a–c). In addition, EX527 treatment suppressed PD-induced translocation of Parkin from the cytoplasm to mitochondria (Fig. 3d–f). These findings indicate that PD induces Parkin-mediated mitophagy via activation of SIRT1.

### Role of mitophagy in the protective effect of PD against SI-AKI

**Fig. 4:**
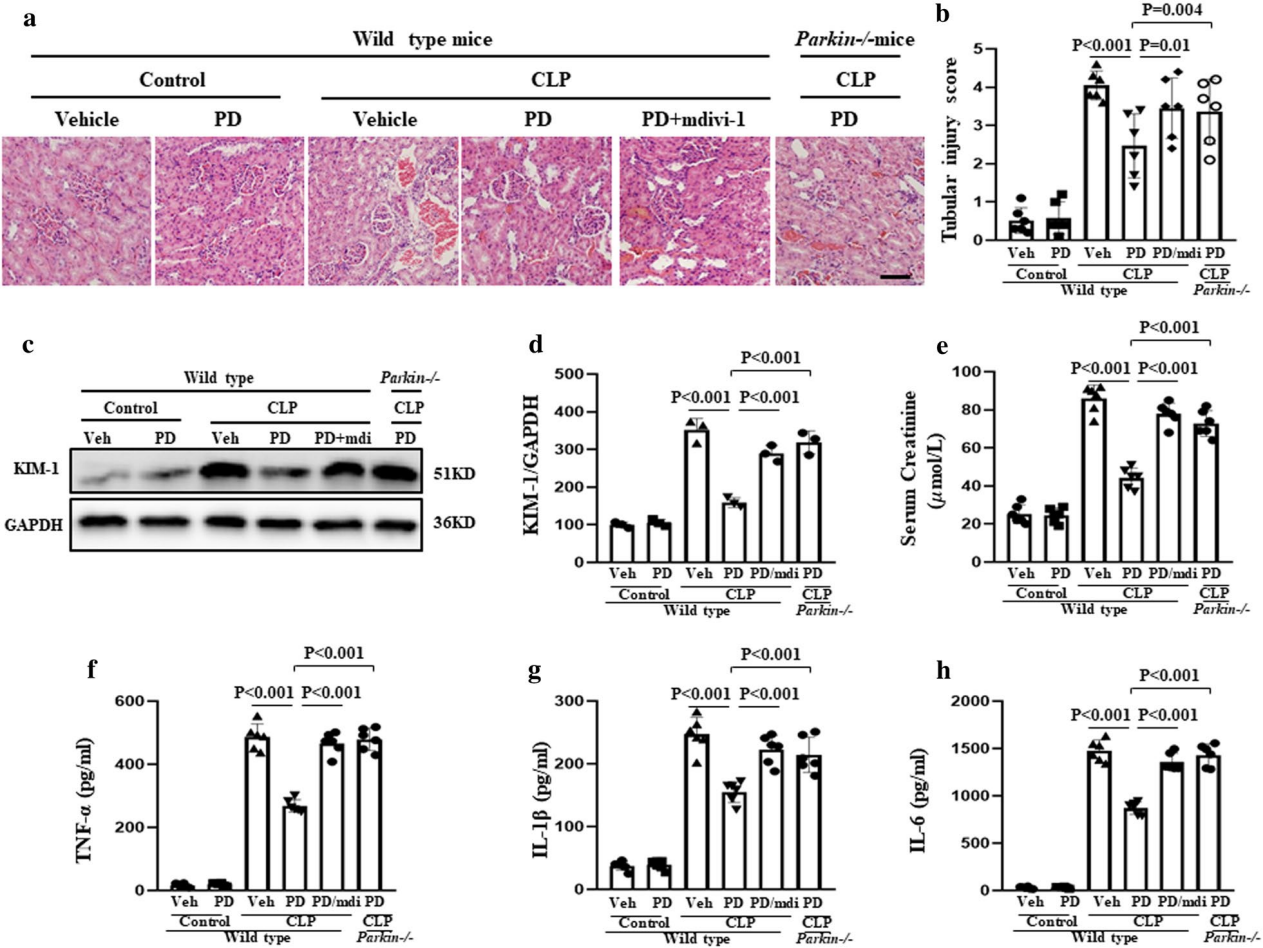
Role of mitophagy in the protective effect of PD against kidney injury in SI-AKI. The mice were subjected to CLP and administered vehicle, PD (30 mg/kg), or PD plus mdivi-1 (3 mg/kg), which is an inhibitor of mitophagy. Parkin−/− mice were subjected to CLP and treated with PD (30 mg/kg). The mice were sacrificed at 12 h following CLP. **a** H&E staining of the renal cells was performed for histological analysis (×400). Scale bar: 500 μm. **b** The tubular injury score was calculated based on visible damage to renal tubule cells. **c** Kim-1 expression was assessed by western blot analysis. Quantification of **d** Kim-1, **e** serum creatinine, **f** serum TNF-α, **g** serum IL-1β, and **h** serum IL-6

Mitophagy was inhibited in SI-AKI model mice by knockout of Parkin or treatment with mdivi-1 (a widely used inhibitor of mitophagy) [18]. The mice were administered vehicle, PD (30 mg/kg), or PD along with mdivi-1 (3 mg/kg). As shown in Fig. 4a, b, PD treatment protected against kidney pathological injury in SI-AKI, which was characterized by the loss of the brush border, cast formation, and vacuolization. In addition, the level of kidney injury molecule-1 (KIM-1), a biomarker of proximal tubular injury, was also significantly decreased with PD treatment (Fig. 4c, d). However, this protective effect of PD was inhibited by mdivi-1 treatment.

Measurement of the serum creatinine concentration (which is an indicator of renal function) showed that PD prevented sepsis-induced renal dysfunction by inhibiting the sepsis-induced increase in serum creatinine. However, the effect of PD on the serum creatinine concentration was blocked by mdivi-1 treatment (Fig. 4e). Moreover, the protective effect of PD against the sepsis-induced increase in serum cytokines, including TNF-α, IL-1β, and IL-6, was inhibited by mdivi-1 treatment (Fig. 4 f–h).

The role of Parkin-mediated mitophagy in the protective effect of PD against SI-AKI was also confirmed by PD treatment of wild-type and Parkin−/− mice. We found that the protective effects of PD against sepsis-induced histopathological deterioration, upregulation of KIM-1, and increase in serum creatinine concentration and serum cytokines were blocked in Parkin−/− mice (Fig. 4).

These findings together suggest that PD treatment provides protection against sepsis-induced kidney injury via Parkin-mediated mitophagy.

### Role of mitophagy in the protective effect of PD against MD and apoptosis

**Fig. 5:**
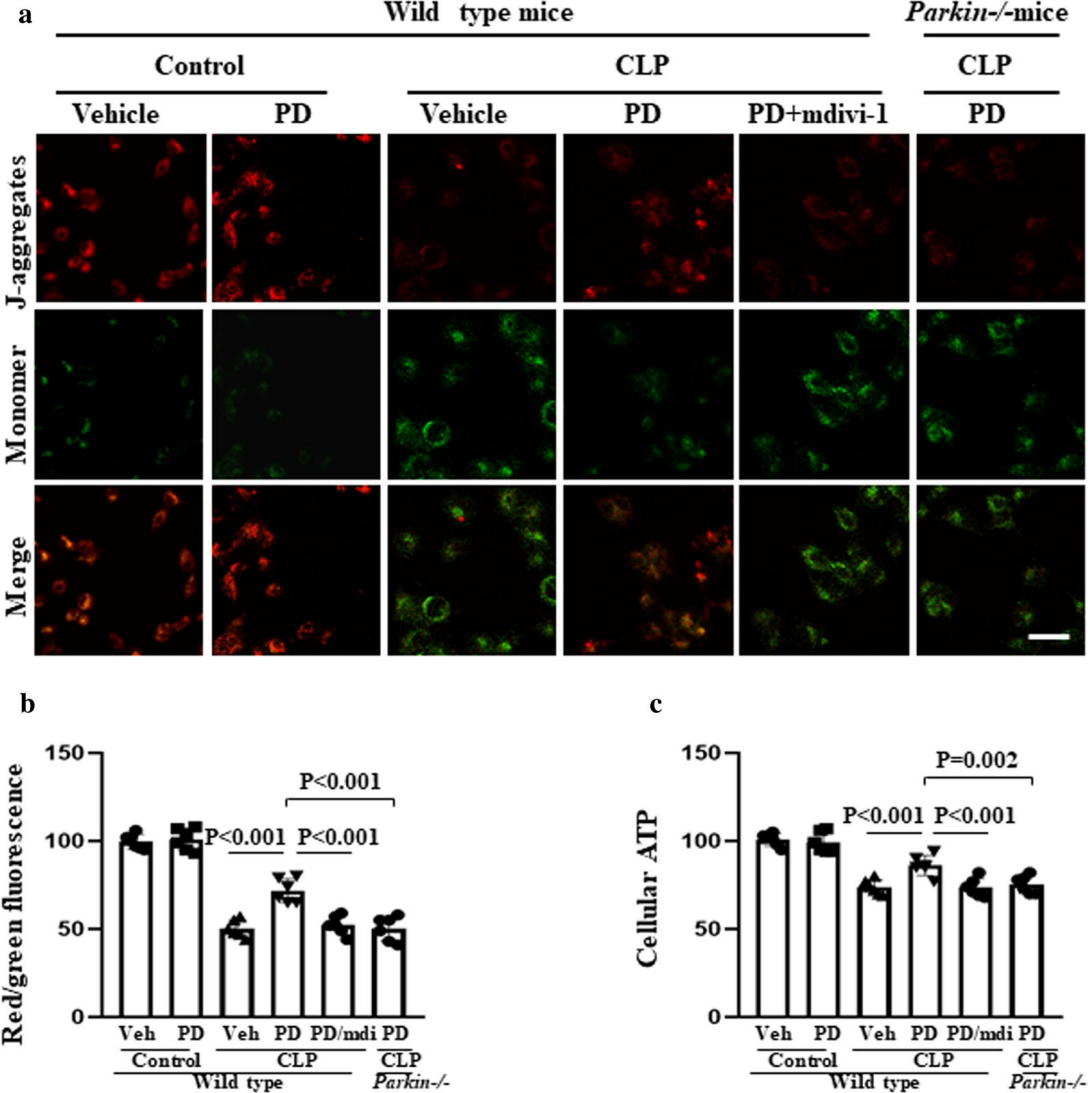
Role of mitophagy in the protective effect of PD against sepsis-induced MD. The mice were subjected to CLP and administered vehicle, PD (30 mg/kg), or PD plus mdivi-1 (3 mg/kg). Parkin−/− mice were subjected to CLP and administered PD (30 mg/kg). The mice were sacrificed at 12 h after CLP. **a** RTECs were isolated and stained with JC-1 to measure mitochondrial membrane potential (MMP) using a laser confocal-scanning microscope. Scale bar: 50 μm. **b** Quantification of the intracellular red and green fluorescence emitted by JC-1. **c** The cellular ATP levels of RTECs were measured by a luciferase-based assay

We found an increase in MMP depolarization and a reduction in cellular ATP levels in SI-AKI model mice. These features are indicative of MD, but these effects were alleviated by PD treatment (Fig. 5). However, the addition of mdivi-1 treatment abrogated these beneficial effects of PD treatment. Furthermore, when wild-type and Parkin−/− mice with SI-AKI were treated with PD, an increase in MMP depolarization and reduction in cellular ATP levels were observed in the Parkin−/− mice. These findings indicate that the protective effect of PD was mediated by Parkin-dependent mitophagy.

**Fig. 6:**
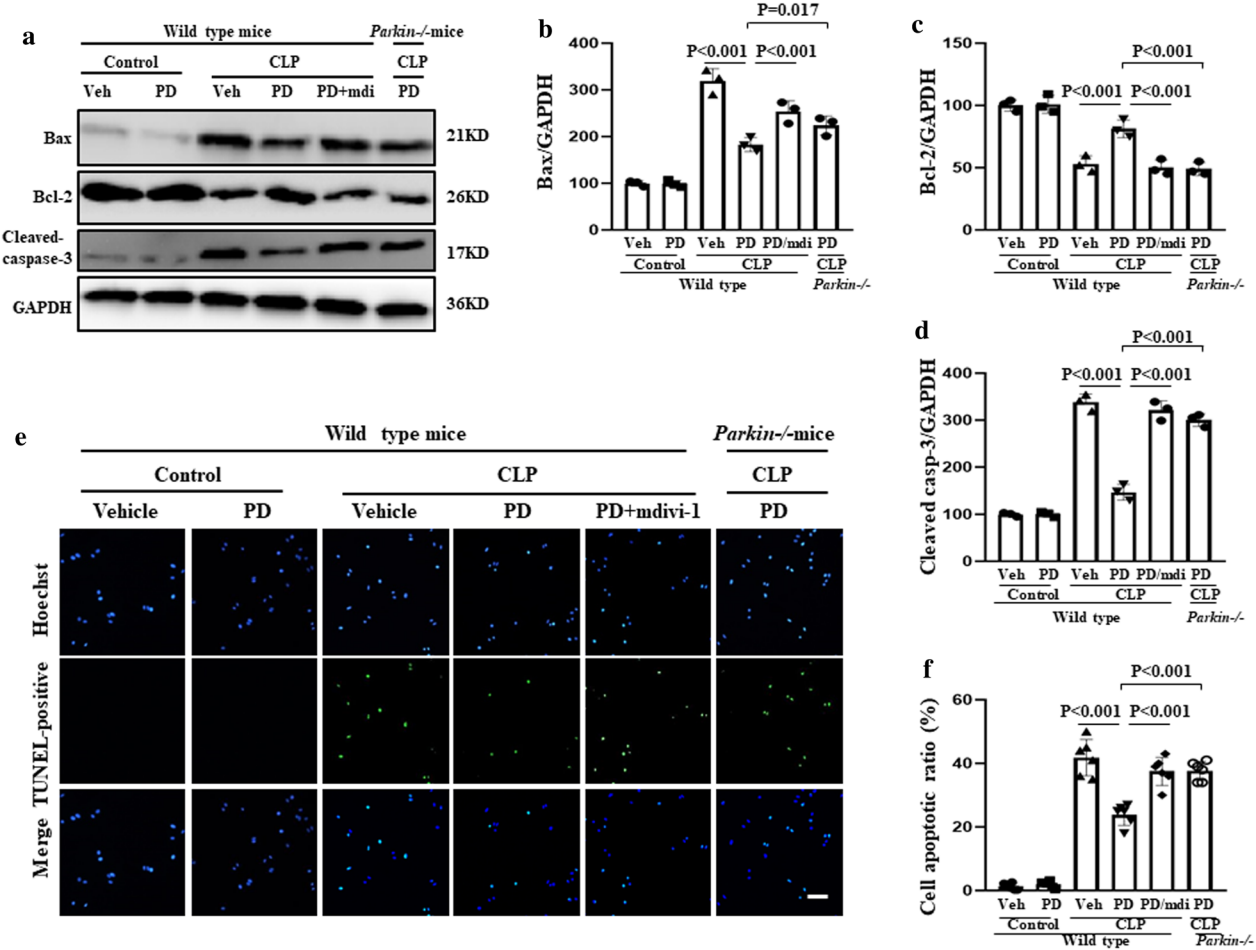
Role of mitophagy in the protective effect of PD against sepsis-induced apoptosis. The mice were subjected to CLP and administered vehicle, PD (30 mg/kg), or PD plus mdivi-1 (3 mg/kg). Parkin−/− mice were subjected to CLP and administered PD (30 mg/kg). The mice were sacrificed at 12 h after CLP. **a** The expression of Bax, Bcl-2 and cleaved caspase-3 in the kidney was assessed by western blot analysis. Quantification of **b** Bax, **c** Bcl-2 and **d** cleaved caspase-3. **e** Apoptosis in isolated RTECs was assessed by TUNEL staining. Scale bar: 100 μm. **f** Quantification of the number of TUNEL-positive cells per field

Mitochondria mediate apoptosis and are involved in AKI [22]. Therefore, we examined the effects of PD-mediated mitophagy on mitochondria-dependent apoptosis. We found that the apoptosis rate and expression of Bax and cleaved caspase-3 increased and Bcl-1 expression decreased in the SI-AKI model mice; these changes were reversed with PD treatment (Fig. 6). However, these protective effects of PD were inhibited by mdivi-1 treatment and Parkin knockout. Thus, PD may also provide protection against mitochondria-dependent apoptosis via Parkin-mediated mitophagy in SI-AKI.

Together, these results suggest that PD restores MD and inhibits mitochondria-dependent apoptosis via Parkin-mediated mitophagy in SI-AKI.

### Role of mitophagy in the protective effect of PD against NLRP3 activation

**Fig. 7:**
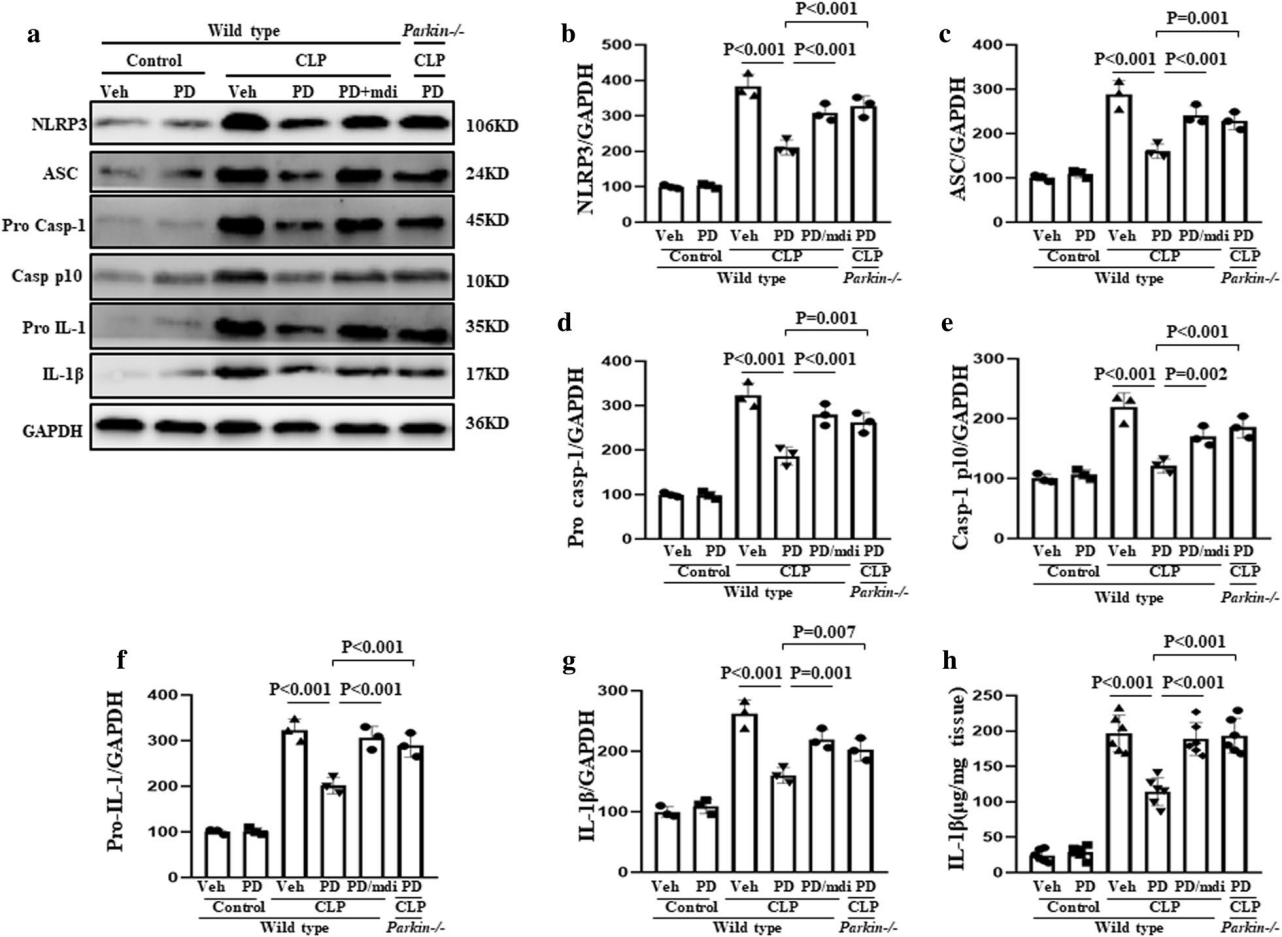
Role of mitophagy in the protective effect of PD against sepsis-induced NLRP3 activation. The mice were subjected to CLP and administered vehicle, PD (30 mg/kg), or PD along with mdivi-1 (3 mg/kg). Parkin−/− mice were subjected to CLP and administered PD (30 mg/kg). The mice were sacrificed at 12 h after CLP. **a** The expression of NLRP3, ASC, pro-caspase-1, caspase-1 p10, pro-IL-1 and IL-1β was measured by western blot analysis. Quantification of **b** NLRP3, **c** ASC, **d** pro-caspase-1, **e** caspase-1 p10, **f** pro-IL-1, and **g** IL-1β. **h** IL-1β in the kidney was measured by ELISA

**Fig. 8:**
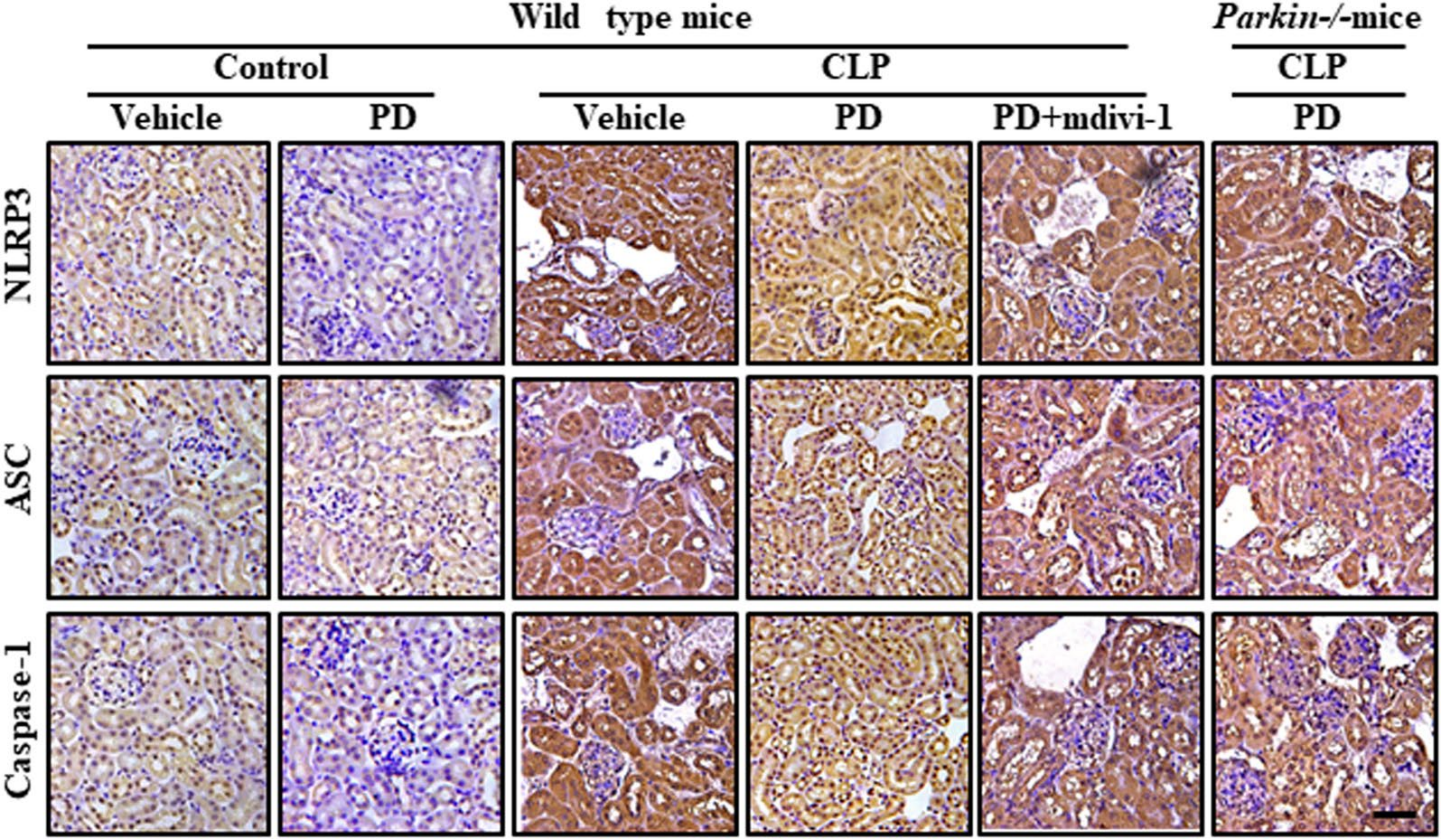
Role of mitophagy in the protective effect of PD against sepsis-induced NLRP3 activation. The mice were subjected to CLP and administered vehicle, PD (30 mg/kg), or PD along with mdivi-1 (3 mg/kg). Parkin−/− mice were subjected to CLP and administered PD (30 mg/kg). The mice were sacrificed at 12 h after CLP. The expression of NLRP3, ASC and Caspase-1 was quantified by immunohistochemical analysis (×400). Scale bar: 500 μm

The components of the NLRP3 inflammasome are NLRP3, apoptosis-associated speck-like protein containing a CARD (ASC) and pro-caspase-1. Activation of the NLRP3 inflammasome results in the initiation of an inflammatory response via caspase-1 activation, which results in the maturation and secretion of the pro-inflammatory cytokine IL-1 [23]. In the present study, NLRP3, ASC, caspase-1, and IL-1β expression in kidney lysate was examined as an indicator of activation of the NLRP3 inflammasome in SI-AKI. Elevated expression of NLRP3, ASC, caspase-1 P10, and IL-1β was observed, indicating that the NLRP3 inflammasome was activated (Fig. 7). However, PD treatment significantly downregulated the expression of these inflammasome proteins. This inhibitory effect of PD was blocked by mdivi-1 treatment and Parkin knockout. These findings were confirmed by immunohistochemical experiments, in which NLRP3 ASC and caspase-1 expression was upregulated in the untreated SI-AKI mice and downregulated in the PD-treated SI-AKI mice (Fig. 8). Furthermore, mdivi-1 treatment and Parkin knockout blocked the PD-mediated downregulation of NLRP3 ASC and caspase-1 expression.

Taken together, these findings indicate that PD negatively regulates NLRP3 activation via Parkin-dependent mitophagy in SI-AKI.

## Discussion

**Fig. 9:**
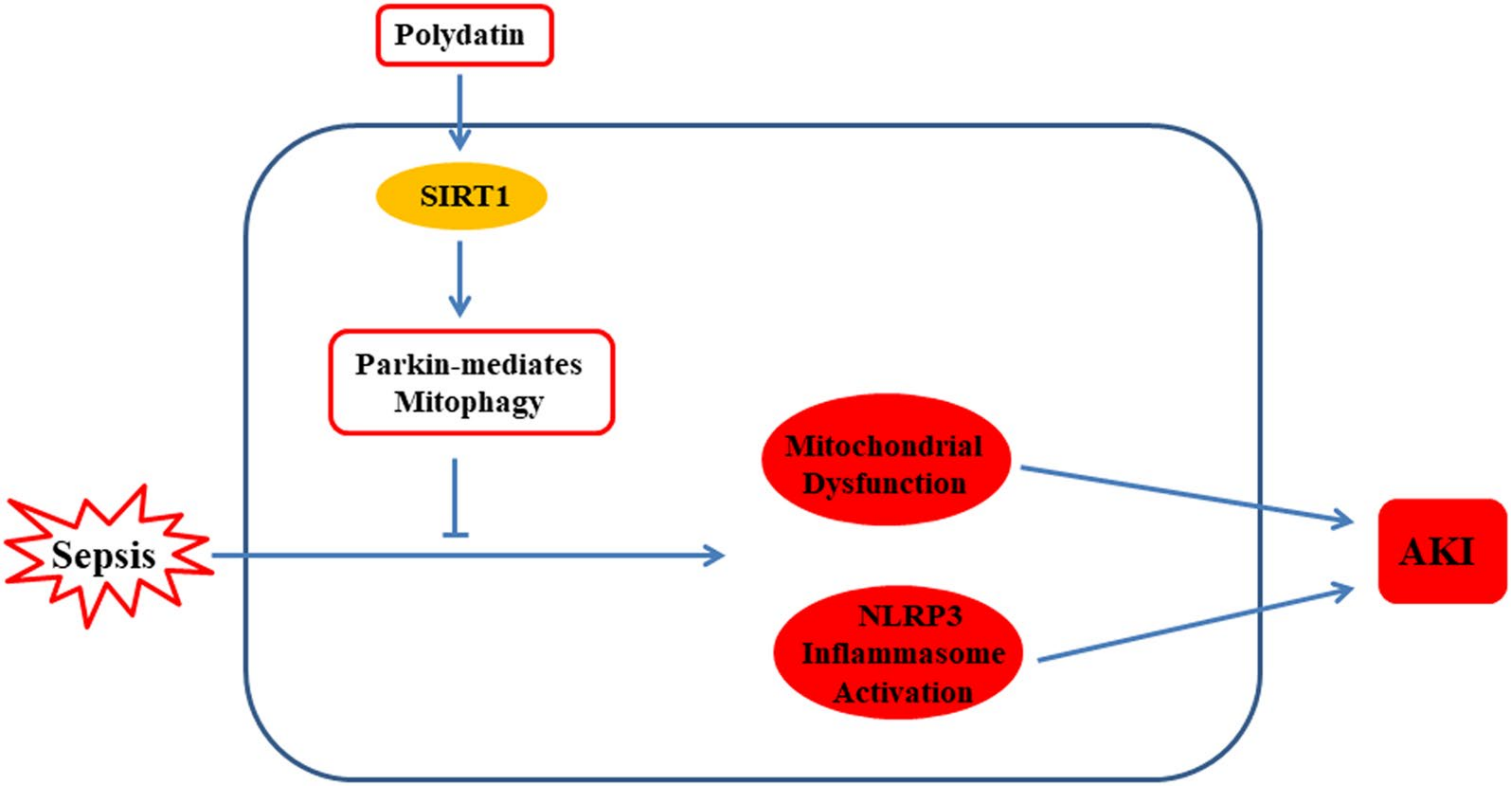
PD upregulated Parkin-dependent mitophagy by activating SIRT1; PD protected against mitochondrial dysfunction and NLRP3 inflammasome activation by upregulating Parkin-dependent mitophagy in acute kidney injury

In the present study, we investigated the role and mechanism of Parkin-mediated mitophagy in the protective effect of PD in a mouse model of SI-AKI (Fig. 9). Our findings show that PD significantly reduced mitochondrial mass via upregulation of mitophagy. Additionally, the findings indicate that Parkin is involved in the mechanism underlying PD-mediated mitophagy. These findings are in agreement with those of our previous study, in which we reported that PD activates Parkin-mediated mitophagy in acute lung injury [18].

The association between SIRT1 and mitophagy has been previously reported [21, 24]. For example, SIRT1 signalling participates in grape-derived antioxidant-induced mitophagy and cardioprotection [25]. In agreement with these reported findings, we demonstrated that PD protects against MD in AKI by activating SIRT1 [26]. Furthermore, in the current study, we investigated the role of SIRT1 in PD-induced mitophagy in SI-AKI model mice. We found that inhibition of SIRT1 by EX527, which is a SIRT1-specific inhibitor, blocked the PD-induced loss of mitochondrial mass and translocation of Parkin; thus, PD protects against SI-AKI by upregulating Parkin-mediated mitophagy via activation of SIRT1. In the future, it would be interesting to explore the possibility of using SIRT1 as a treatment target in SI-AKI. In addition, we have not clarified the mechanism by which SIRT1 regulates Parkin-dependent mitophagy in the present study. Therefore, the molecular mechanism of mediating mitophagy by sirtuins will be the focus of our future work.

We have previously demonstrated that PD attenuates MD and the inflammatory response in a septic rat model of AKI [1]; however, the underlying mechanism was not clarified in the previous study. In ischaemia–reperfusion-induced AKI, PARK2-mediated mitophagy is important for the quality control of mitochondria, survival of tubular cells, and renal function [27]. Additionally, upregulation of Parkin-mediated mitophagy prevents cisplatin-induced mitochondrial dysfunction and cell injury in RTECs [9]. Therefore, in the present study, we investigated the role of mitophagy in the protective effects of PD against SI-AKI. For this purpose, we treated SI-AKI model mice with mdivi-1, an inhibitor of mitophagy [18], and studied the effects of PD in Parkin-knockout mice. The results showed that inhibition of mitophagy blocked the protective effect of PD against SI-AKI, renal dysfunction, and the upregulated expression of KIM-1 and serum cytokines, including TNF-α, IL-1β, and IL-6. These findings indicate that PD treatment alleviates SI-AKI by upregulating Parkin-dependent mitophagy. Our results are consistent with a previous study showing that Parkin-mediated mitophagy plays a protective role in septic AKI [28].

MD plays an important role in several diseases, including AKI [19, 29, 30]. Additionally, MD is often associated with the triggering of mitophagy pathways, which eliminate dysfunctional or impaired mitochondria [6, 31]. Therefore, in the present study, we investigated whether Parkin-mediated mitophagy participates in PD-induced protection against SI-AKI via its effect on MD. We found that inhibition of mitophagy with mdivi-1 and Parkin knockout blocked the PD-induced protective effects against MD in RTECs. In addition, the mitochondria-regulated intrinsic apoptotic signalling pathway plays an important role in AKI [22]. Accordingly, we found that PD-induced alleviation of sepsis-induced apoptosis in RTECs was also inhibited by mdivi-1 treatment and Parkin knockout. Thus, our present findings indicate that PD-induced Parkin-mediated mitophagy protects against MD and mitochondria-associated apoptosis. These mechanisms should be studied further to explore potential treatment targets.

The important role of the NLRP3 inflammasome in renal inflammation has been demonstrated in several renal disease models, including AKI [14, 23]. Tang et al. [23] reported that downregulation of NLRP3 inflammasome activation protects the kidney against ischaemia/reperfusion injury. Additionally, Xiao et al. [12] demonstrated that NLRP3 activation mediated by oxidative stress plays an important role in susceptibility to AKI in diabetes models. Studies have also shown that the NLRP3 inflammasome contributes to sepsis [32, 33]. Based on these findings, it can be hypothesised that the NLRP3 inflammasome is a potential target for AKI treatment. The protective effects of PD against NLRP3 activation in kidney disease have been reported [34]; however, the mechanism underlying this effect has yet to be fully elucidated. Here, we demonstrated that inhibition of Parkin-dependent mitophagy blocked the negative regulation of NLRP3 activation by PD. This suggests that PD inhibits NLRP3 activation via Parkin-dependent mitophagy in SI-AKI. Consistent with this finding, some studies have proposed that MD is closely associated with NLRP3 inflammasome activation [17]. According to one such study, mitochondrial DNA released by damaged mitochondria and mitochondrial ROS are thought to trigger NLRP3 activation [35, 36]. Recently, the role of mitophagy in the prevention of NLRP3 inflammasome activation has also been reported [17]. Li et al. [37] reported that Parkin-mediated mitophagy impairs antiviral immunity by suppressing the mtROS-NLRP3 axis. Additionally, Zhang et al. [38] reported that impairment in mitophagy induces NLRP3 inflammasome activation during disease progression from nonalcoholic fatty liver to nonalcoholic steatohepatitis. To the best of our knowledge, this is the first study to report PD-induced NLRP3 inflammasome inactivation as a molecular mechanism of mitophagy in SI-AKI. Increasing studies have reported that inflammasome formation-induced activation of pyroptosis plays an important role in inflammatory diseases, including SI-AKI [39–41]. In the future, the effects of PD and sirtuins on pyroptosis should be explored further to identify more treatment targets for SI-AKI.

## Conclusion

In conclusion, the findings of this study suggest that PD protects against MD and NLRP3 inflammasome activation in SI-AKI by activating Parkin-dependent mitophagy. With regard to the underlying mechanism, the findings indicate that PD upregulates Parkin-dependent mitophagy via activation of SIRT1. The findings of this study are promising, and they need to be confirmed in human patients to explore the possibility of using PD as a therapeutic agent for SI-AKI.

## Data Availability

The datasets used and/or analysed in this study will be made available by the corresponding author (Tao Li) upon reasonable request.

## Abbreviations

SI-AKI: Sepsis-induced acute kidney injury
PD: Polydatin
MD: Mitochondria dysfunction
SIRT1: Silent mating type information regulation 2 homolog-1
LC3: Microtubule-associated protein 1 light chain 3
TUNEL: Terminal deoxynucleotidyl transferase dUTP nick-end labelling
NS: Normal saline
Drp1: Dynamin-related protein 1
TOM20: Translocase of outer mitochondrial membrane 20
TIM23: Translocase of inner mitochondrial membrane 23
MMP: Mitochondrial membrane potential
H&E: Haematoxylin and eosin
KIM-1: Kidney injury molecule 1
NLRP3: NACHT, LRR, and PYD domains-containing protein 3
JC-1: 5,5ʹ,6,6ʹ-Tetrachloro-1,1ʹ,3,3ʹ-tetraethyl-imidacarbocyanine iodide
ROS: Reactive oxygen species
Caspase: Cysteinyl aspartate specific proteinase
PGC-1α: Peroxisome proliferator-activated receptor-γ coactivator-1α
mTFA: Mitochondrial transcription factor A
P62/SQSTM1: Sequestosome 1
IL: Interleukin
ASC: Apoptosis-associated speck-like protein
CLP: Caecal ligation and puncture
GAPDH: Glyceraldehyde-3-phosphate dehydrogenase
COX: Cytochrome c oxidase
Mdiv-1: Mitochondrial division inhibitor
